# A Simple and Reliable Setup for Monitoring Corrosion Rate of Steel Rebars in Concrete

**DOI:** 10.1155/2014/525678

**Published:** 2014-01-12

**Authors:** Shamsad Ahmad, Mohammed Abdul Azeem Jibran, Abul Kalam Azad, Mohammed Maslehuddin

**Affiliations:** Civil and Environmental Engineering Department, King Fahd University of Petroleum and Minerals, P.O. Box 1403, Dhahran 31261, Saudi Arabia

## Abstract

The accuracy in the measurement of the rate of corrosion of steel in concrete depends on many factors. The high resistivity of concrete makes the polarization data erroneous due to the Ohmic drop. The other source of error is the use of an arbitrarily assumed value of the Stern-Geary constant for calculating corrosion current density. This paper presents the outcomes of a research work conducted to develop a reliable and low-cost experimental setup and a simple calculation procedure that can be utilised to calculate the corrosion current density considering the Ohmic drop compensation and the actual value of the Stern-Geary constants calculated using the polarization data. The measurements conducted on specimens corroded to different levels indicate the usefulness of the developed setup to determine the corrosion current density with and without Ohmic drop compensation.

## 1. Introduction

The rate of reinforcement corrosion, commonly expressed as corrosion current density, *I*_corr_, is a quantitative indicator of the damage caused to the steel rebars due to their corrosion. Such information is of great importance for detecting corrosion initiation at early stages and for predicting the time to initiation of corrosion-induced cracking and residual strength of a corroding structure and hence it's remaining service-life [1]. The nondestructive test methods generally utilised for measuring corrosion rate include Tafel plot, linear polarization resistance, electrochemical noise, A.C. impedance, and electrical resistance. The main advantages of electrochemical techniques include sensitivity to low corrosion rates, short experimental duration, and well-established theoretical understanding. On the other hand, the gravimetric weight loss measurement is a destructive technique for determining the average rate of corrosion [2].

The linear polarization resistance method (LPRM) is mostly used for measuring the *I*_corr_ of the rebar embedded in concrete, as it is nondestructive and nonperturbative. Furthermore, the LPRM is suitable for use in the laboratory and in the field while other methods are only suitable for use in the laboratory. The setup used in LPRM consists of a working electrode, a counter-electrode, a reference electrode, and a DC power supply unit; usually a potentiostat or galvanostat is utilised for this purpose.

The potentiostat or galvanostat is connected to the rebar (the working electrode), counter-electrode, and the reference electrode. In the potentiostatic mode, the potential difference between the working and the counter-electrode is kept constant and the resulting current is measured. In the galvanostatic mode the current flowing between the counter-electrode and the working electrode is kept constant and the resulting potential of the working electrode is measured. For both modes the potential and current are plotted and the slope of this curve, denoted as the resistance to polarization, *R*_*p*_, is calculated, and this value is used to calculate the *I*_corr_ utilising the well-known Stern-Geary equation [3]. The main difference in determining the *R*_*p*_ for big-size laboratory specimens or in situ measurements, compared to small-size laboratory specimens, is the geometrical arrangement of the counter electrode. In laboratory studies, the concrete specimen containing the steel rebar as working electrode, the counter-electrode, and the reference electrode are immersed in a sodium chloride solution which promotes a uniform distribution of the polarizing current on the steel rebars. In the case of a big-size specimen or in the in situ measurement, a disk-shaped counter-electrode (having a central hole to accommodate the reference electrode) is placed on a water-soaked sponge kept on the surface of the concrete specimen/structure. The reference electrode is placed in the central hole of the counter-electrode.

The *I*_corr_ measurements in large-size laboratory concrete specimens or in in situ structures using the LPRM have been a challenging problem because of various practical difficulties encountered in the application of the available test methods and instruments resulting in a significant error in the estimation of the corrosion rate. The major sources of errors include failure to adequately correct for the Ohmic drop resulting from the relatively high electrical resistivity of concrete; lack of information on the area of the rebar that is actually being polarized; and the lack of precise value of the Stern-Geary constant used for the calculation of the corrosion rate. For an improved or better estimation of *I*_corr_, the correct measurement of *R*_*p*_ taking into consideration the Ohmic drop compensation and the area of rebar actually polarized and also the determination of the actual value of the Stern-Geary constant, *B*, using the values of Tafel slopes are essential. For this purpose, the aspects that need to be considered are adequate compensation for Ohmic drop, verification of the range of linearity of the polarization around *E*_corr_, selection of an optimum polarization response time, careful interpretation of the *R*_*p*_ value particularly when the steel is in a passive state, solving the problem of uncertainty of the rebar surface actually polarized by the applied electric signal using a suitable method, and the determination of the values of the Tafel slopes (for calculating the actual value of *B*) using the polarization data [4–15].

A simple experimental setup and calculation procedure were reported earlier by Ahmad and Bhattacharjee [8] considering the accuracy by having a provision of Ohmic drop compensation and determination of the precise value of the Stern-Geary constant. However, the performance of the setup developed by Ahmad and Bhattacharjee [8] was not evaluated for its application for measuring a wide range of very low to very high *I*_corr_. Commercial setups are also available in recent times for measuring reinforcement corrosion rate but the accuracy of the commercially available setups needs to be verified for their applications to reinforcement corrosion rate. Furthermore, the commercial setups have not been shown to be reliable when Ohmic drop compensation option was utilized. The objective of the present study was to develop a simple low-cost setup for measuring *I*_corr_ by making a minor modification in the original setup of Ahmad and Bhattacharjee [8]. The performance of the developed setup was evaluated for determining its capability to measure *I*_corr_ in all ranges with and without Ohmic drops compensation.

## 2. Development of a Setup and a Calculation Procedure to Estimate *I*_corr_

### 2.1. Circuitry of the Setup

**Figure 1:**
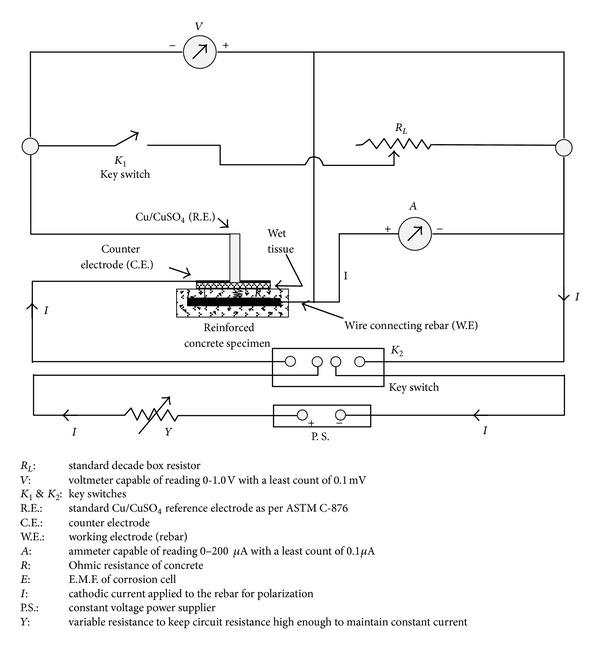
Experimental setup for generating the data pertaining to corrosion potential, Ohmic resistance, and polarization resistance (modified version of the setup proposed earlier by Ahmad and Bhattacharjee [8]).

**Figure 2:**
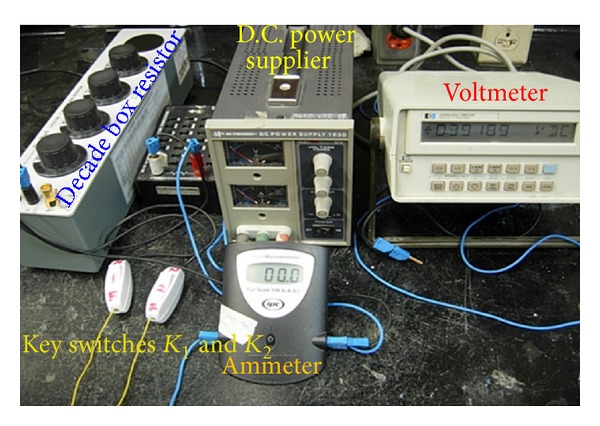
Photograph showing components of the developed setup.

Slightly modified version of the circuitry proposed earlier by Ahmad and Bhattacharjee [8] is shown in Figure 1. The only modification made in the circuitry was the connection of the wire connecting rebar (working electrode) to the positive terminal of the voltmeter in addition to the connection through the ammeter. This change made it possible to have stable readings in ammeter during polarization, particularly at low current intensities. The circuitry is based on the linear polarization resistance method and it is integrated in a way that the data can be generated to calculate the Ohmic resistance of concrete and Tafel slopes required for calculating *I*_corr_ more accurately. The part of the integrated circuitry for measuring the half-cell potential using a Cu/CuSO_4_ electrode (CSE) as a reference electrode is similar to that specified by ASTM C 876-99 [16]. The part of the integrated circuitry for determining the Ohmic resistance is based on the principle of determining the internal resistance of a cell [17]. The part of the integrated circuitry for the determination of the apparent polarization resistance is based on the galvanostatic technique [18]. The counter-electrode (C.E.) for applying the polarizing current was made of stainless steel having a width of 25 mm and thickness of 3 mm with a central hole to accommodate the reference electrode (R.E.). A photograph of the components of the developed setup is shown in Figure 2.

### 2.2. Data Generation Using the Developed Setup

#### 2.2.1. Preparation of the Test Points

Before using the setup for carrying out measurements, there should be good physical contact between the reference electrode tip and the concrete surface to avoid poor electrolytic contact of the reference electrode with the specimen. For this purpose, the prewetting of the surface points under test is done through a tissue paper soaked with water and allowed to be there for 20–30 minutes so that the moisture can soak in before the initiation of the test.

Referring to Figure 1 and the earlier work published by Ahmad and Bhattacharjee [8], the procedure for measurement of half-cell potential, that is, corrosion potential (*E*_corr_) and generation of data required to determine Ohmic resistance (*R*), and polarization resistance (*R*_*p*_) are as follows.

#### 2.2.2. Corrosion Potential (*E*_corr_)

Keeping key switches *K*_1_ and *K*_2_ off, the corrosion potential *E*_corr_ is recorded allowing a sufficient response time of 30–60 seconds for measurements to stabilize. If the corrosion potential is low, the voltmeter reading is not stable and fluctuates; consequently, the reading after 30–60 seconds waiting period should be considered as the representative value.

#### 2.2.3. Data for Ohmic Resistance (*R*)

For determining the Ohmic resistance (*R*), different value of resistances (*R*_*L*_) is set in the standard decade box resistor and *R*_*L*_ versus *V*_*L*_ data are generated keeping key switch *K*_1_on and key switch *K*_2_ off. The switch *K*_1_ permits a standard decade box resistor to be connected momentarily, whenever a voltage reading is desired, thus avoiding excessive current drain over a prolonged period of time. The terminal voltage of the cell, under load, *V*_*L*_, is given by the voltmeter reading, when the key switch, *K*_1_, is closed. At least 10 values of *V*_*L*_ are recorded by setting different values of *R*_*L*_ in increasing order with a gap of 10 to 15 s between two consecutive readings.

#### 2.2.4. Data for Polarization Resistance (*R*_*p*_)

For determining the polarization resistance (*R*_*p*_), a cathodic polarizing current, *I*, is applied and the resulting potential *V* (which is more negative than *E*_corr_) is recorded by keeping key switch *K*_1_ off throughout the experiment and key switch *K*_2_ on. The current is applied in steps until the maximum value of the overvoltage, *ε* (value of potential by which *E*_corr_ is shifted as a result of polarization), is reached, which is usually 10 to 20 mV for the polarization curve to be in the linear range, with the help of a variable resistor, *Y*, which helps to keep the resistance of the circuit high enough to maintain a constant current. Initially, a cathodic current of 2 *μ*A is applied and then the second current step is 4 *μ*A, the third 6 *μ*A, and so forth. After allowing a response time of 30 seconds at each current step the steady voltmeter reading is recorded. After polarizing, a significant fluctuation in the voltage of rebars with low corrosion is noted and hence a response time of 30 seconds is commonly used to note the voltmeter reading.

### 2.3. Calculation Procedure for Corrosion Parameters

The Ohmic resistance (*R*), polarization resistance (*R*_*p*_), Tafel slopes (*β*_*a*_ and *β*_*c*_), Stern-Geary constant (*B*), and corrosion current density (*I*_corr_) are determined, as follows.

#### 2.3.1. Determination of Ohmic Resistance (*R*)

The 1/*R*_*L*_ and 1/*V*_*L*_ values are plotted keeping 1/*R*_*L*_ on the *x*-axis and 1/*V*_*L*_ on the *y*-axis. The slope and *y*-axis intercept of the best-fit straight line joining these points are noted down. The ratio of the value of the slope to the value of intercept gives the value of the Ohmic resistance, *R*. The value of the Ohmic resistance can be utilised for compensating the Ohmic drop mathematically.

#### 2.3.2. Determination of Polarization Resistance (*Rp*)

With the help of the recorded polarization data (*I* versus *V* values), the polarized potential (*E*) can be determined as *E* = *V* (without Ohmic drop compensation) or *E*′ = *V* − *I* · *R* (Ohmic drop compensation), where *R* is the Ohmic resistance obtained in the previous step. Using the value of *E*, the overvoltage (*ε*) can be determined as *ε* = *E* − *E*_corr_, where *E*_corr_ is the measured value of the corrosion potential (without applying polarization current). The *I* versus **ε** values are plotted and a straight line is best-fitted. The slope of the best-fitted straight line is taken as *R*_*p*_.

#### 2.3.3. Determination of Tafel Slopes (*β*_*a*_ and *β*_*c*_) and Stern-Geary Constant (*B*)

Instead of assuming the value of the Stem-Geary constant, *B*, as 26 mV for actively corroding reinforcement and 52 mV for passive reinforcement [19], the Tafel slopes *β*_*a*_ and *β*_*c*_ should be determined utilising the polarization data for determining its accurate value. The values of *β*_*a*_ and *β*_*c*_ can be determined by best-fitting the polarization data into the polarization equation, as follows [20]:

(1) $$\begin{array}{c} 2.3R_{p}I_{i}=\frac{\beta_{a}\beta_{c}}{\beta_{a}+\beta_{c}}\left[\exp\left(\frac{2.3\varepsilon_{i}}{\beta_{a}}\right)-\exp\left(-\frac{2.3\varepsilon_{i}}{\beta_{c}}\right)\right]. \end{array}$$

The search method can be used to determine *β*_*a*_ and *β*_*c*_, in which several possible combinations of the values of *β*_*a*_ and *β*_*c*_ can be tried within their minimum and maximum values of 120 mV to 240 mV corresponding to *B*-value in the range of 26 mV to 52 mV [19]. The final value of *β*_*a*_ and *β*_*c*_ can be taken corresponding to the minimum value of the sum of squares of the differences of left-hand side and right-hand side values of (1) for *i* number of the *I* versus *ε* values within the linear polarization range. The minimization of the sum of error squares can be carried out using *Excel-Solver* and the optimum values of *β*_*a*_ and *β*_*c*_ can be determined satisfying the constraints: *β*_*a*_ and *β*_*c*_ should be between 120 and 240 mV. Once the values of *β*_*a*_ and *β*_*c*_ are determined, the Stern-Geary constant, *B*, can be determined using (2) [3]

(2) $$\begin{array}{c} B=\frac{\beta_{a}\beta_{c}}{2.3\left(\beta_{a}+\beta_{c}\right)}. \end{array}$$

#### 2.3.4. Determination of Corrosion Current Density (*I*_corr_)

*I*_corr_ can be determined using the calculated values of *R*_*p*_, *B*, and surface area of the steel rebar, *A*_*s*_, from (3) [3]

(3) $$\begin{array}{c} I_{\text{corr}}=\frac{B}{R_{p}A_{s}}. \end{array}$$

## 3. Experimental Program

### 3.1. Test Specimens

**Figure 3:**
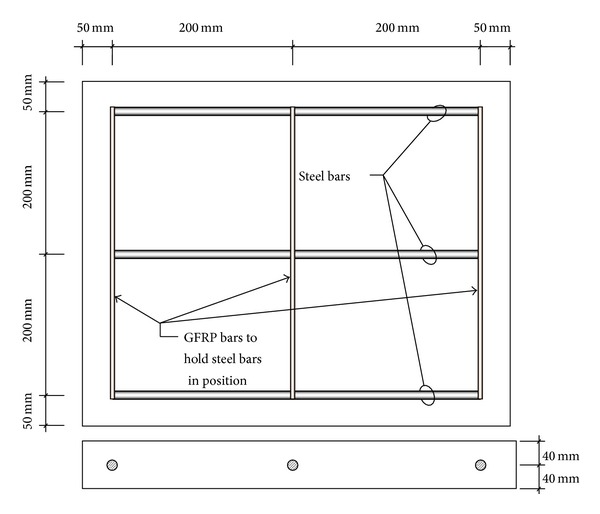
Details of reinforced concrete slab specimens.

**Figure 4:**
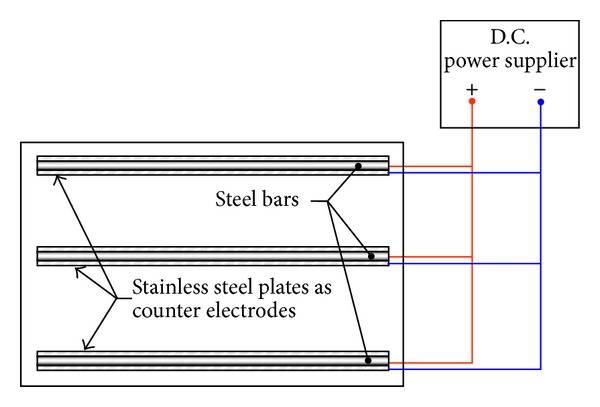
Setup for corroding the steel rebars.

Fifteen reinforced concrete specimens reinforced with three 12 mm diameter bars in each specimen were prepared to assess the efficacy of the developed setup in measuring reinforcement corrosion. The details of the concrete specimens are shown in Figure 3. In Figure 3, three steel bars embedded in concrete are represented by three thick horizontal lines. The three thin vertical lines represent the GFRP bars embedded in concrete to hold the steel bars in correct positions during casting. Steel rebars embedded in the slab specimens were corroded to varying degree using the impressed current technique. The positive terminal of a D.C. power supply was connected to the steel rebar and the negative terminal was connected to the stainless steel plates (25 mm wide and 3 mm thick strips used as counter electrodes), placed over the concrete surface corresponding to the line of steel rebars, each having a surface area of 158.33 cm^2^, as shown in Figure 4. Different degrees of reinforcement corrosion were obtained by varying the duration of the impressed current in the range of 27 to 280 hours.

Since the slab specimens were corroded by low intensity impressed current for simulating natural corrosion, the maximum corrosion rate measured in such specimens was found to be less than 1 *μ*A/cm^2^. In order to examine the usefulness of the developed setup for measuring varying intensities of corrosion (low, medium, and high), another different batch of 13 specimens, naturally corroded under chloride exposures for a period of about five years, was considered in which the *I*_corr_ was in the range of 1 *μ*A/cm^2^ to 10 *μ*A/cm^2^.

### 3.2. *I*_corr_ Measurements

**Figure 5:**
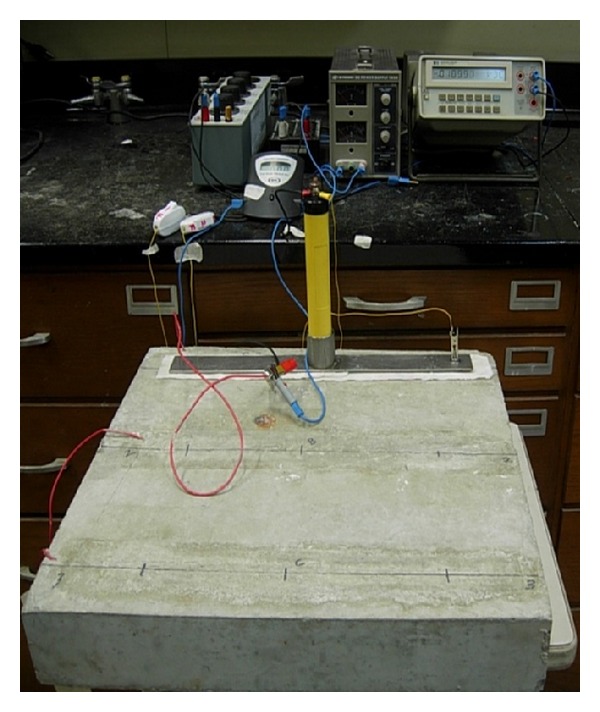
*I*_corr_ measurement using developed setup.

The developed setup was utilized to measure *I*_corr_ in the test specimens prepared and corroded in accelerative and natural manners. A photograph of the corrosion measurement using the developed setup is shown in Figure 5. The white layer under the steel plate is water-soaked tissue paper placed to improve the electrolytic contact of the reference electrode with the specimen. The *I*_corr_ values were calculated with and without *IR* compensation.

### 3.3. Calculations for Determining *I*_corr_

**Figure 6:**
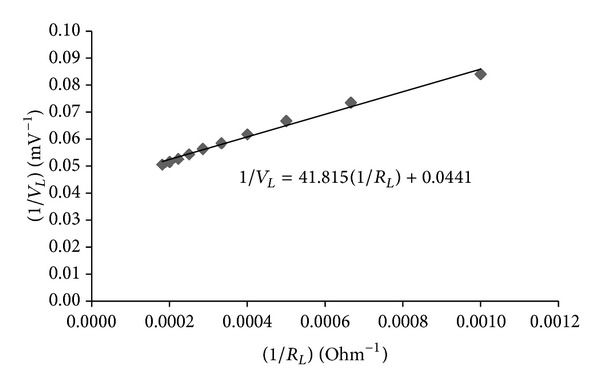
Plot of 1/*R*_*L*_ versus 1/*V*_*L*_ values for obtaining Ohmic resistance, *R*.

Table 1 shows a set of typical data for calculating the Ohmic resistance, *R*. By plotting the 1/*R*_*L*_ versus 1/*V*_*L*_, a best-fit straight line is obtained, as shown in Figure 6. The ratio of the slope (41.815) and intercept (0.0441) gives the value of *R* as 948 Ohm.

**Figure 7:**
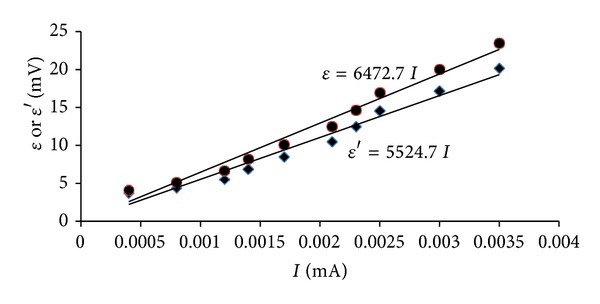
Plot of *I* versus *ε* values for obtaining polarization resistance, *R*_*p*_ and *R*_*p*_′.

A typical set of the polarization data along with the measured value of *E*_corr_ and calculated value of *R* are presented in Table 2. The values of over voltage *ε* and *ε*′ for without and with Ohmic drop compensation, respectively, are also shown in Table 2. Using the *I* versus *ε* values, the best-fitted linear polarization curves for without and with Ohmic drop compensation are also plotted, as shown in Figure 7. The slopes of the best-fitted straight lines are taken as the polarization resistance values as *R*_*p*_ = 6473 Ohm (without Ohmic drop compensation) and *R*_*p*_′ = 5525 Ohm (with Ohmic drop compensation). After obtaining the values of polarization resistance, Tafel slopes can be calculated by best-fitting equation (1) using *Excel-Solver*. The calculations of the Tafel slopes as well as Stern-Geary constant are presented in Table 2. Using the calculated values of polarization resistance, Stern-Geary constant, and surface area of the steel rebar (158.33 cm^2^), the corrosion current density can be calculated using (3), as follows: *I*_corr_ = 46.57 × 10^3^/(6473 × 158.33) = 0.045 *μ*A/cm^2^ (without *IR* drop compensation) and *I*_corr_ = 45.69 × 10^3^/(5525 × 158.33) = 0.052 *μ*A/cm^2^ (with *IR* drop compensation).

Three sets of *I*_corr_ measurements were carried out on each of 15 slab specimens corroded in accelerative way and one set of *I*_corr_ measurement was conducted on each of 13 cylindrical specimens corroded naturally. In this way a total of 58 sets of *I*_corr_ values were determined with and without *IR* drop compensation utilizing the developed setup and calculation procedure, as presented in Table 3. The measured values of *I*_corr_ were found to be in the range of 0.026 *μ*A/cm^2^ to 3.38 *μ*A/cm^2^ without Ohmic drop compensation and 0.03 *μ*A/cm^2^ to 14.3 *μ*A/cm^2^ with Ohmic drop compensation. The *I*_corr_ measurements in a wide range of very low to very high values, using the developed setup, have confirmed that the setup is capable of measuring corrosion rates of any degree, from very low to very high.

## 4. Results and Discussions

### 4.1. Variation of Polarization Trend with Degree of Corrosion

**Figure 8:**
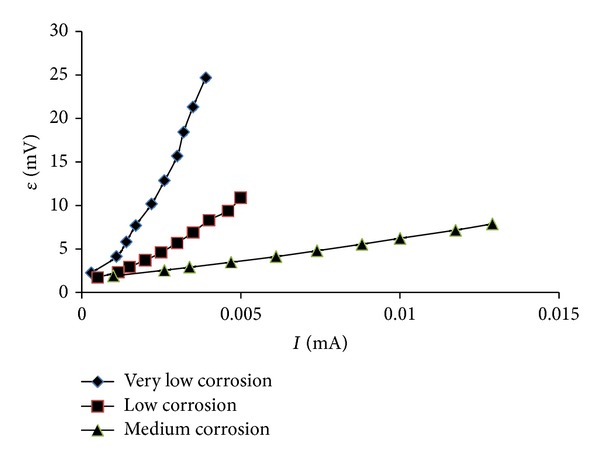
Polarization curves for specimens with very low, low, and medium corrosion.

In order to show the variation of the polarization trend with the variation in the *I*_corr_ from low to high, polarization data (*I* versus *ε* values) obtained for three different specimens typically having very low, low, and medium degree of corrosion were plotted, as shown in Figure 8. It can be seen from the data in Figure 8 that the linearity of the polarization curves is more with an increase in the degree of corrosion. Further, in case of very low corrosion, the overpotential is very high at a very low value of the polarizing current. For a higher degree of corrosion, the requirement for polarizing current increases even for a relatively very small overpotential.

**Figure 9:**
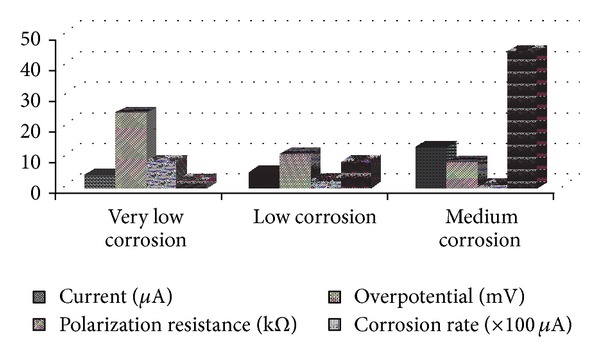
Comparison of *I*, *ε*, *R*_*p*_, and *I*_corr_ for very low, low, and medium corrosion.

Figure 9 shows a comparison of *I*, *ε*, *R*_*p*_, and *I*_corr_ values for varying intensity of corrosion. It can be observed that the requirement for polarizing current *I* increases while the resulting overpotential, *ε*, decreases with an increase in the degree of corrosion. Further, as expected, the polarization resistance, *R*_*p*_, decreased while the corrosion rate, *I*_corr_, increased with an increase in the degree of corrosion.

### 4.2. Correlation between *I*_corr_Values Measured with and without *IR* Drop Compensation

**Figure 10:**
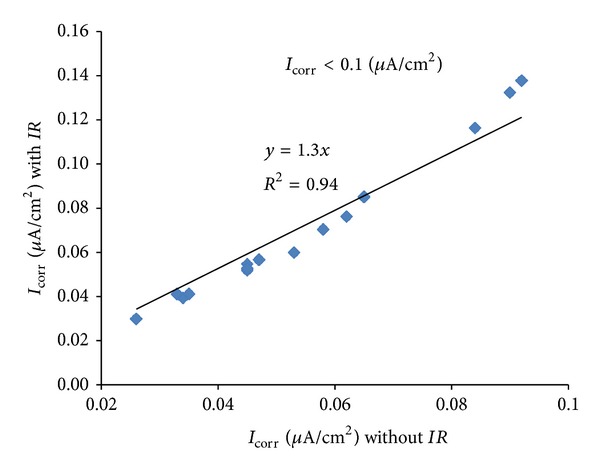
Correlation between *I*_corr_ with and without *IR* compensation for *I*_corr_ < 0.1 *μ*A/cm².

**Figure 11:**
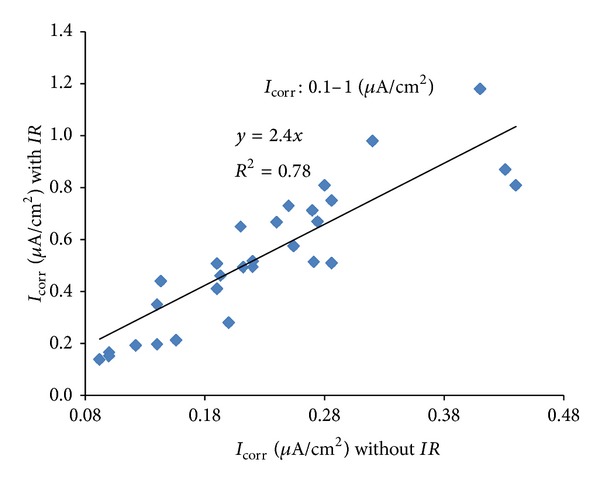
Correlation between *I*_corr_ with and without *IR* compensation for *I*_corr_ 0.1 to 1.0 *μ*A/cm².

**Figure 12:**
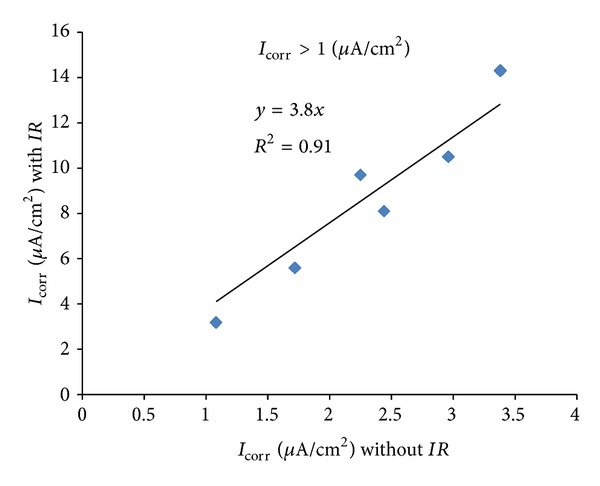
Correlation between *I*_corr_ with and without *IR* compensation for *I*_corr_ > 1 *μ*A/cm².

The *I*_corr_ values presented in Table 3 were utilised for developing correlation between *I*_corr_ values measured with and without Ohmic drop compensation. For this purpose, the data were classified into three groups for different ranges of the degree of corrosion, as follows: (i) low corrosion (*I*_corr_ < 0.1 *μ*A/cm²); (ii) medium corrosion (*I*_corr_ = 0.1 to 1.0 *μ*A/cm²); and (iii) high corrosion (*I*_corr_ > 1 *μ*A/cm²). The plots of *I*_corr_ with and without *IR* compensation for low, medium, and high corrosion are shown in Figures 10, 11, and 12, respectively. The equations correlating between *I*_corr_ with and without *IR* compensation are presented in Table 4. These data show that the effect of Ohmic drop increases with an increase in the intensity of corrosion. The error in *I*_corr_ due to Ohmic drop is only 30% in case of low degree of corrosion against an error of around 250% in case of medium degree of corrosion while it is 400% in the case of high degree of corrosion. The increase in the effect of Ohmic drop is due to the higher requirement for polarizing current for the specimens having a higher degree of corrosion, as shown in Figure 9.

The data developed in this study have indicated that the *I*_corr_ can be determined without *IR* compensation with a fair degree of accuracy in specimens with low degree of corrosion (*I*_corr_ < 0.1 *μ*A/cm²). However, *IR* compensation must be considered in determining medium to high *I*_*corr*_ (*I*_corr_ > 0.1 *μ*A/cm²). Alternatively, the *I*_corr_ can be determined without *IR* compensation and the actual values can be obtained by multiplying with a coefficient of 2.4 for medium corrosion (*I*_corr_ = 0.1 to 1.0 *μ*A/cm^2^) and 3.8 for high corrosion (*I*_corr_ > 1 *μ*A/cm²). It should be noted that the experimental setup and calculation procedure developed in the present study are useful in situations where *I*_corr_ measurement with *IR* compensation is not possible. Many of the *I*_corr_ measuring equipment commercially available in the market do not provide stable readings if the *IR* compensation is utilized.

The measurements using the proposed setup are possible for a normal thickness of concrete cover provided for reinforced concrete members. However, the effect of thickness of concrete cover on accuracy of the measurements should be considered in a separate study. The proposed setup and calculation procedure can be used in laboratory as well as in the field for measuring the corrosion current density of steel bars embedded in concrete with *IR* compensation. For the field applications, the rebars should be located using suitable equipment such as a cover meter for rightly placing the reference and counter electrodes and also for making connections with the rebars.

## 5. Conclusions

Based on the outcomes of the present study, the following conclusions are made.

1. A reliable experimental setup and a simple calculation procedure were developed that may be used to determine the *I*_corr_ over a wide range of values (0.1 *μ*A/cm^2^ to more than 10 *μ*A/cm^2^). The *I*_corr_ values can be determined with and without *IR* compensation.
2. The *I*_corr_ measured with *IR* compensation was more than that measured without *IR* compensation. The effect of Ohmic drop was found to be low when the corrosion rate was low but its effect increased with an increase in the intensity of corrosion. The increase in Ohmic drop is due to higher requirement for polarizing current for the specimens with a high degree of corrosion.
3. The equations developed in this study can be utilized to correct the values of *I*_corr_ determined without Ohmic drop compensation to obtain the *I*_corr_ actual values. *I*_corr_ can be determined with a fair degree of accuracy in specimens with low intensity of corrosion (*I*_corr_ < 0.1 *μ*A/cm²) without *IR* compensation. However, in specimens with medium to high corrosion a correction factor of 2.4 to 3.8 is required.

## Figures and Tables

**Table 1:** Sample calculation for *R*.

Specimen ID: AC-4 with *E*_corr_ = 57 mV; *R* = 948 Ohm			
*R*_*L*_ (Ohm)	*V*_*L*_ (mV)	1/*R*_*L*_	1/*V*_*L*_
1000	11.9	0.0010	0.08
1500	13.6	0.0007	0.07
2000	15.0	0.0005	0.07
2500	16.2	0.0004	0.06
3000	17.1	0.0003	0.06
3500	17.8	0.0003	0.06
4000	18.4	0.0003	0.05
4500	19.0	0.0002	0.05
5000	19.4	0.0002	0.05
5500	19.8	0.0002	0.05

**Table 2:** Sample calculation for *R*_*p*_, *β*_*a*_, *β*_*c*_, and *B*.

Specimen ID: AC-4 with *E*_corr_ = 57 mV (CSE) and *R* = 948 Ohm							
*I*	*E* = *V*	*E*′ = *V* − *IR*	*ε* = *E* − *E*_corr_	*ε*′ = *E*′ − *E*_corr_	*R*_*p*_ and *R*_*p*_′	Square of the error for (1) (without *IR* drop)	Square of the error for (1) (with *IR* drop)
(mA)	(mV)	(mV)	(mV)	(mV)	(Ohm)		
0.0004	61.08	60.70	4.08	3.70	*R*_*p*_ = 6473 *R*_*p*_′ = 5525	11.530	11.534
0.0008	62.12	61.36	5.12	4.36		0.034	0.031
0.0012	63.65	62.51	6.65	5.51		7.138	7.047
0.0014	65.20	63.87	8.20	6.87		4.551	4.460
0.0017	67.10	65.48	10.10	8.48		5.288	5.160
0.0021	69.47	67.47	12.47	10.47		8.431	8.222
0.0023	71.62	69.43	14.62	12.43		1.076	1.007
0.0025	73.90	71.53	16.90	14.53		1.244	1.301
0.003	77.00	74.15	20.00	17.15		0.410	0.191
0.0035	80.50	77.18	23.50	20.18		1.135	1.128

Minimization of the sum of the squares of the error for best fitting (1) with the help of the Excel-Solver using constraints as follows: *β*_*a*_ and *β*_*c*_ ≥ 120 mV but ≤240 mV; *B* ≥ 26 mV but ≤52 mV.						Min. sum = 40.836 at *β*_*a*_ = 240 mV and *β*_*c*_ = 193 mV Therefore, from (2) *B* = 46.57 mV	Min. sum = 40.085 at *β*_*a*_ = 240 mV and *β*_*c*_ = 187 mV Therefore, from (2) *B* = 45.69 mV

**Table 3:** *I*_corr_ (with and without *IR* compensation) measured using developed setup.

Specimen ID	*I*_corr_ without *IR* (*μ*A/cm²)	*I*_corr_ with *IR* (*μ*A/cm²)
AC-1	0.026	0.030
AC-2	0.034	0.039
AC-3	0.033	0.041
AC-4	0.045	0.052
AC-5	0.084	0.116
AC-6	0.058	0.070
AC-7	0.035	0.041
AC-8	0.062	0.076
AC-9	0.053	0.060
AC-10	0.065	0.085
AC-11	0.100	0.165
AC-12	0.047	0.057
AC-13	0.045	0.052
AC-14	0.045	0.055
AC-15	0.045	0.053
AC-16	0.090	0.132
AC-17	0.140	0.196
AC-18	0.122	0.192
AC-19	0.286	0.509
AC-20	0.065	0.085
AC-21	0.156	0.212
AC-22	0.240	0.667
AC-23	0.143	0.441
AC-24	0.100	0.152
AC-25	0.090	0.111
AC-26	0.210	0.650
AC-27	0.190	0.508
AC-28	0.212	0.493
AC-29	0.440	0.809
AC-30	0.320	0.979
AC-31	0.286	0.751
AC-32	0.140	0.349
AC-33	0.092	0.138
AC-34	0.254	0.576
AC-35	0.220	0.495
AC-36	0.250	0.969
AC-37	0.280	0.809
AC-38	0.410	1.180
AC-39	0.193	0.461
AC-40	0.274	0.670
AC-41	0.190	0.411
AC-42	0.271	0.514
AC-43	0.250	0.730
AC-44	0.220	0.517
AC-45	0.270	0.712
NC-1	0.200	0.280
NC-2	0.250	0.350
NC-3	0.210	0.350
NC-4	0.240	0.380
NC-5	0.560	0.660
NC-6	0.431	0.870
NC-7	0.560	0.850
NC-8	2.250	9.700
NC-9	2.960	10.500
NC-10	2.440	8.100
NC-11	3.380	14.300
NC-12	1.720	5.600
NC-13	1.082	3.180

AC: accelerated corrosion; NC: normal corrosion.

**Table 4:** Relation between *I*_corr_ without and with Ohmic drop for various degrees of corrosion.

*I*_corr_ (*μ*A/cm²)	Degree of corrosion	Relation between *I*_corr_ measured without and with Ohmic drop
<0.1	Low	(*I*_corr_) with *IR* = 1.3 (*I*_corr_) without *IR*
0.1 to 1	Medium	(*I*_corr_) with *IR* = 2.4 (*I*_corr_) without *IR*
>1	High	(*I*_corr_) with *IR* = 3.8 (*I*_corr_) without *IR*

## Symbols

*A*_*s*_: Surface area of the steel rebar exposed to corrosion
*B*: Stern-Geary constant
*E* and *E*′: Polarized potential without and with Ohmic drop compensation, respectively
*E*_corr_: Corrosion potential
*I*: Cathodic current applied to the rebar for polarization
*I*_corr_: Corrosion current density
*IR*: Ohmic drop compensation
*R*: Ohmic resistance of concrete
*R*_*p*_ and *R*_*p*_′: Polarization resistance without and with Ohmic drop compensation, respectively
*V*_*L*_: Potential drop corresponding to the resistance set in the standard decade box resistor, *R*_*L*_
*β*_*a*_ and *β*_*c*_: Anodic and cathodic Tafel slopes, respectively
*ε* and *ε*′: Overpotential without and with Ohmic drop compensation, respectively.
